# Clinical and functional outcomes for risk‐appropriate treatments for prostate cancer

**DOI:** 10.1002/bco2.288

**Published:** 2023-09-12

**Authors:** Tenaw Tiruye, Michael O'Callaghan, Kerry Ettridge, Kim Moretti, Alex Jay, Braden Higgs, Kerry Santoro, Ganessan Kichenadasse, Kerri Beckmann

**Affiliations:** ^1^ Cancer Epidemiology and Population Health Research Group, Allied Health and Human Performance University of South Australia Adelaide Australia; ^2^ Public Health Department Debre Markos University Debre Markos Ethiopia; ^3^ South Australian Prostate Cancer Clinical Outcomes Collaborative Adelaide Australia; ^4^ Flinders Health and Medical Research Institute Flinders University Adelaide Australia; ^5^ Discipline of Medicine University of Adelaide Adelaide Australia; ^6^ Flinders Medical Centre Bedford Park Australia; ^7^ Health Policy Centre South Australian Health and Medical Research Institute Adelaide Australia; ^8^ School of Public Health University of Adelaide Adelaide Australia; ^9^ Discipline of Surgery University of Adelaide Adelaide Australia; ^10^ Department of Radiation Oncology Royal Adelaide Hospital Adelaide Australia; ^11^ Southern Adelaide Local Health Network Adelaide Australia

**Keywords:** biochemical recurrence, health outcomes, prostate cancer, quality of life, survival

## Abstract

**Objectives:**

To describe real‐world clinical and functional outcomes in an Australian cohort of men with localised prostate cancer according to treatment type and risk category.

**Subjects and methods:**

Men diagnosed from 2008 to 2018 who were enrolled in South Australian Prostate Cancer Clinical Outcomes Collaborative registry—a multi‐institutional prospective clinical registry—were studied. The main outcome measures were overall survival, cancer‐specific survival, decline in functional outcomes, biochemical recurrence and transition to active treatment following active surveillance. Multivariable adjusted models were applied to estimate outcomes.

**Results:**

Of the 8513 eligible men, majority of men (46%) underwent radical prostatectomy (RP) followed by external beam radiation therapy with or without androgen deprivation therapy (EBRT +/− ADT) in 22% of the cohort. Five‐year overall survival was above 91%, and 5‐year prostate cancer‐specific survival was above 97% in the low‐ and intermediate‐risk categories across all treatments. Five‐year prostate cancer‐specific survival in the active surveillance group was 100%. About 37% of men with high‐risk disease treated with RP and 17% of men treated with EBRT +/− ADT experienced biochemical recurrence within 5 years of treatment. Of men on active surveillance, 15% of those with low risk and 20% with intermediate risk converted to active treatment within 2 years. The decline in urinary continence and sexual function 12 months after treatment was greatest among men who underwent RP while the decline in bowel function was greatest for men who received EBRT +/− ADT.

**Conclusion:**

This contemporary real‐world evidence on risk‐appropriate treatment outcomes helps inform treatment decision‐making for clinicians and patients.

## 1 INTRODUCTION

In 2021, prostate cancer accounted for 23% of all new cancer cases and 12% of all cancer deaths among Australian males, imposing a significant burden on the healthcare system.^1^ While prognosis is generally very favourable for men with localised disease who undergo radical treatments (i.e. prostatectomy or radiotherapy), the impact on physical functioning can be substantial.^2^ Side effects following radical prostatectomy (RP) include erection and ejaculation problems, urinary incontinence, reduced penis length and loss of fertility, while radiation therapies may lead to urinary problems such as frequency, urgency, burning/discomfort and haematuria; bowel problems such as increased frequency, urgency and rectal bleeding; erection and ejaculation problems; and fatigue.^3^ Active surveillance is regarded as a safe alternative for men with low‐risk (indolent) disease to reduce morbidity associated with radical therapies.^4^ For men on active surveillance, anxiety about potential disease progression may impact their quality of life or lead them to unnecessarily opt for active treatment.^5^ Men who require androgen deprivation therapy (ADT) will invariably experience hormonal imbalances and be at increased risk of developing additional comorbidities such as cardiovascular disease and diabetes.^6^

Risk categorisation, based on biopsy grade, prostate‐specific antigen (PSA) levels and clinical stage at diagnosis, is the major determinant of appropriate treatment options for men with prostate cancer.^7^ However, most treatment guidelines specify a range of risk‐appropriate treatment options, with choice being guided by patient and physician preferences on survival benefits and impacts on physical functioning.^8^ Evidence from ‘real‐world data’, reflecting actual clinical practice, can be a useful guide for decision‐making.^9^

This study describes clinical and functional outcomes following different primary treatment modalities in a contemporary Australian cohort using outcomes data from the South Australian Prostate Cancer Clinical Outcome Collaborative (SA‐PCCOC) registry. We evaluated survival, biochemical recurrence, conversion to active treatment during active surveillance and impact on sexual, urinary, bowel and hormonal function.

## 2 SUBJECTS AND METHODS

This study was ethically approved by University of South Australia Human Research Ethics Committee (protocol: 203716) and the Southern Adelaide Clinical Human Research Ethics Committee (LNR/22/SAC/10). Prostate cancer outcomes were assessed using data from SA‐PCCOC. Established in 1998, SA‐PCCOC is a disease‐specific, population‐wide multi‐institutional registry that prospectively collects diagnostic, treatment, clinical outcome data and patient‐reported functional outcomes for men with prostate cancer in South Australia. SA‐PCCOC contributes core data to the binational Prostate Cancer Outcomes Registry for Australia and New Zealand (PCOR‐ANZ). SA‐PCCOC collects patient‐reported outcome data at baseline and sequential time points and PSA follow‐up data beyond the 12 months, whereas PCOR‐ANZ only collect patient‐reported outcome measures (PROMs) and PSA data at 12 months post‐treatment. Currently, SA‐PCCOC has enrolled over 19 000 men from both public and private treatment centres and captures 90% of newly diagnosed cases in South Australia.

Our study cohort included men with prostate cancer diagnosed between 1 January 2008 and 31 December 2018, who consented to be in the registry. This period was chosen to ensure both currency and adequate follow‐up. Men with distant metastatic prostate cancer or no primary treatment recorded were excluded.

Missing data for diagnostic PSA, Gleason score and clinical stage were imputed to generate the risk categories, using multiply imputed chained equations,^10^ with 10 imputed data sets. Risk category was defined according to National Clinical Cancer Network^11^ (low risk: International Society of Urological Pathology (ISUP) Grade group 1 and PSA < 10 ng/dL and clinical‐stage T1–T2a; intermediate risk: ISUP Grade group 2–3, or PSA ≥ 10–20 ng/dL, or stage T2b–T2c; and high risk: ISUP Grade group 4–5, or PSA > 20 ng/dL, or stage T3a–T4). Primary treatment was defined as the initial or most definitive treatment within 12 months of diagnosis. Treatment groups included RP, external beam radiotherapy with or without ADT (EBRT +/− ADT), brachytherapy (high dose and low dose), ADT alone, active surveillance and watchful waiting. While active surveillance involves close ongoing monitoring and subsequent treatment by radiation or surgery with the intent to cure, watchful waiting involves occasional monitoring and treatment for symptoms if they arise, usually by hormone therapy, without the intention to cure the cancer. Men who had (neo)adjuvant ADT (*n* = 488), that is, those who had EBRT within 12 months of starting ADT, were grouped with EBRT.

Covariates included age at diagnosis, year of diagnosis and socio‐economic advantage. Area‐level measures of socio‐economic advantage were derived from the 2016 Australian Bureau of Statistics socio‐economic index for areas scores, based on an individual's residential postcode.^12^

The following outcomes, which are endorsed by the International Committee on Health Outcome Measure Standardisation^13^ were assessed: (1) overall survival; (2) prostate cancer‐specific survival; (3) biochemical recurrence following radical therapies; (4) transition to active treatment following active surveillance; and (5) clinically significant changes in physical functioning in relation to sexual dysfunction, urinary incontinence, urinary obstruction, bowel symptoms and hormonal symptoms 12 months after treatment.

Information on date and cause of death was obtained from SA‐PCCOC registry via the SA births, deaths and marriages registry and SA Cancer Registry, which undertakes routine linkages with state and national death indexes. Biochemical recurrence was derived from follow‐up PSA data, and defined according to treatment‐specific criteria for RP (two consecutive PSA measures >0.2 ng/dL^14^) and radiotherapy (2.0 ng/dL above the PSA nadir following radiotherapy^15^). Functional outcomes were measured at baseline and 12‐month post‐treatment using Expanded Prostate Cancer Index Composite (EPIC)‐26 domain scores.^16^ EPIC‐26 provides scores for five functional domains that range from 0 to 100, with higher scores indicating better function. We applied Skolarus et al.'s suggested values (10 points for sexual function, 6 points for urinary continence, 5 points for urinary obstruction and 4 points for both bowel and hormonal function) to define clinically significant declines in function at 12‐month post‐treatment.^17^

### 2.1 Statistical analyses

Flexible survival models were executed to estimate all‐cause and disease‐specific survival probabilities for all treatments, the probability of developing biochemical recurrence following radical treatments and the probability of transitioning to active treatment at 2 and 5 years for men on active surveillance, as described by Lambert et al.^18^ Separate subgroup analyses were undertaken for each risk category, with adjustment for age, socio‐economic advantage and year of diagnosis, followed by post‐estimation prediction to estimate survival probabilities for each treatment in each relevant risk category. Follow‐up time was calculated from the date of primary treatment to the date of the event, date of death or censoring date (14 June 2021). Men who died from other causes were censored from prostate cancer‐specific survival estimation. Active surveillance was excluded from modelling outcomes for high‐risk disease, while ADT alone was excluded from models for low‐risk disease.

For functional outcomes, we used logistic regression models to predict likelihood of experiencing clinically significant decline. Functional outcomes data were not available for all men enrolled in SA‐PCCOC registry. This is due to late notification of their diagnosis (so that baseline surveys were not able to be sent before treatment) or survey non‐response (response rates at each round were ~60%). Inverse probability propensity weighting was used to adjust for any variability between respondents and non‐respondents. The use of propensity weighting to account for missingness has been suggested in previous literature.^19^

Final models were fitted adjusting for inverse probability weight, risk category, age at diagnoses, year of diagnosis and socio‐economic advantage. In these analyses, no adjustments were made for salvage treatments or conversion to active treatment during the 12‐month follow‐up period. Sensitivity analyses in which men who had EBRT within 12 months of RP were excluded (*n* = 76) did not affect the results substantially; hence, we reported outcomes collectively for all men who underwent RP.

For greater equivalence across treatment groups, we reported probabilities of each outcome measure for a 68‐year‐old man (i.e. the average age at diagnosis of the study cohort). All analyses were performed using Stata version 15.0 (StataCorp, College Station, TX, USA).

## 3 RESULTS

In total, 8513 men were included. The mean age of participants at diagnosis was 68 years (SD ± 9). Forty six per cent had undergone RP, 22% had EBRT +/− ADT, 16% initially underwent observant management (active surveillance or watchful waiting), 9% received ADT alone, and 6% had brachytherapy. Summaries of patient characteristics are presented in Table 1.

**TABLE 1:** Characteristic of participants, SA‐PCCOC registry (men diagnosed 2008–2018) (*n* = 8513).

Variables	Categories	RP (*n* = 3945)		EBRT +/− ADT (*n* = 1848)		BT^a^ (*n* = 545)		AS (*n* = 1137)		WW (*n* = 274)		ADT^b^ (*n* = 764)	
		No.	%	No.	%	No.	%	No.	%	No.	%	No.	%
Age (years)	Below 60	1007	25.5	116	6.3	140	25.7	270	23.7	9	3.3	49	6.4
	60–64	935	23.7	197	10.7	132	24.2	229	20.1	13	4.7	83	10.9
	65–70	1318	33.4	437	23.6	157	28.8	339	29.8	42	15.3	118	15.4
	Above 70	685	17.4	1098	59.4	116	21.3	299	26.3	210	76.6	514	67.3
	Mean ± sd	63.9 ± 7.0		71.6 ± 7.4		64.4 ± 7.2		65.2 ± 7.9		76.2 ± 8.3		74.3 ± 9.2	
Risk category	Low	800	20.3	179	9.7	173	31.7	779	68.5	115	42.0	11	1.4
	Intermediate	2277	57.7	878	47.5	308	56.5	233	20.5	98	35.8	168	22.0
	High	751	19.0	704	38.1	33	6.1	19	1.7	32	11.7	569	74.5
	Missing	117	3.0	87	4.7	31	5.7	106	9.3	29	10.6	16	2.1
Diagnostic PSA	<4	415	10.5	83	4.5	54	9.9	208	18.3	38	13.9	28	3.7
	4–10	2407	61.0	699	37.8	352	64.6	673	59.2	102	37.2	135	17.7
	10–20	615	15.6	485	26.2	46	8.4	122	10.7	57	20.8	182	23.8
	>20	142	3.6	242	13.1	4	0.7	13	1.1	20	7.3	336	44.0
	Missing	366	9.3	339	18.3	89	16.3	121	10.6	57	20.8	83	10.9
Gleason score	<=6	1025	26.0	336	18.2	206	37.8	958	84.3	178	65.0	53	6.9
	3 + 4	1476	37.4	481	26.0	214	39.3	108	9.5	53	19.3	106	13.9
	4 + 3	785	19.9	410	22.2	85	15.6	19	1.7	22	8.0	136	17.8
	8	384	9.7	301	16.3	21	3.9	4	0.4	9	3.3	167	21.9
	9–10	226	5.7	261	14.1	7	1.3	0	0.0	6	2.2	267	34.9
	Missing	49	1.2	59	3.2	12	2.2	48	4.2	6	2.2	35	4.6
Level of socio‐economic advantage	Lowest	640	16.2	459	24.8	149	27.3	241	21.2	47	17.2	200	26.2
	Low	724	18.4	426	23.1	119	21.8	209	18.4	74	27.0	164	21.5
	Middle	726	18.4	408	22.1	107	19.6	214	18.8	50	18.2	177	23.2
	High	853	21.6	305	16.5	94	17.2	246	21.6	52	19.0	126	16.5
	Highest	1002	25.4	250	13.5	76	13.9	227	20.0	51	18.6	97	12.7
Year of diagnosis	2008	323	8.2	204	11.0	38	7.0	22	1.9	33	12.0	104	13.6
	2009	341	8.6	221	12.0	38	7.0	51	4.5	39	14.2	104	13.6
	2010	270	6.8	166	9.0	62	11.4	55	4.8	38	13.9	69	9.0
	2011	225	5.7	128	6.9	26	4.8	80	7.0	33	12.0	64	8.4
	2012	227	5.8	164	8.9	28	5.1	106	9.3	34	12.4	61	8.0
	2013	346	8.8	122	6.6	42	7.7	119	10.5	24	8.8	58	7.6
	2014	381	9.7	134	7.3	58	10.6	90	7.9	14	5.1	47	6.2
	2015	434	11.0	160	8.7	70	12.8	92	8.1	14	5.1	53	6.9
	2016	456	11.6	129	7.0	65	11.9	106	9.3	10	3.6	49	6.4
	2017	509	12.9	225	12.2	80	14.7	153	13.5	19	6.9	57	7.5
	2018	433	11.0	195	10.6	38	7.0	263	23.1	16	5.8	98	12.8

*Note*: Men with known metastasis at diagnosis (*n* = 65) were excluded from the analyses.

Abbreviations: ADT, androgen deprivation therapy alone; AS, active surveillance; BT, brachytherapy (low dose and high dose combined); EBRT +/− ADT, external beam radiation therapy with or without ADT; PSA, prostate‐specific antigen; RP, radical prostatectomy; sd, standard deviation; WW, watchful waiting.

^a^ High‐dose‐rate brachytherapy (*n* = 162) was grouped with low‐dose‐rate brachytherapy.

^b^ The most frequently received ADTs were bicalutamide 295 (40%), goserelin 250 (34%) and cyproterone acetate 163 (22%).

**FIGURE 1:**
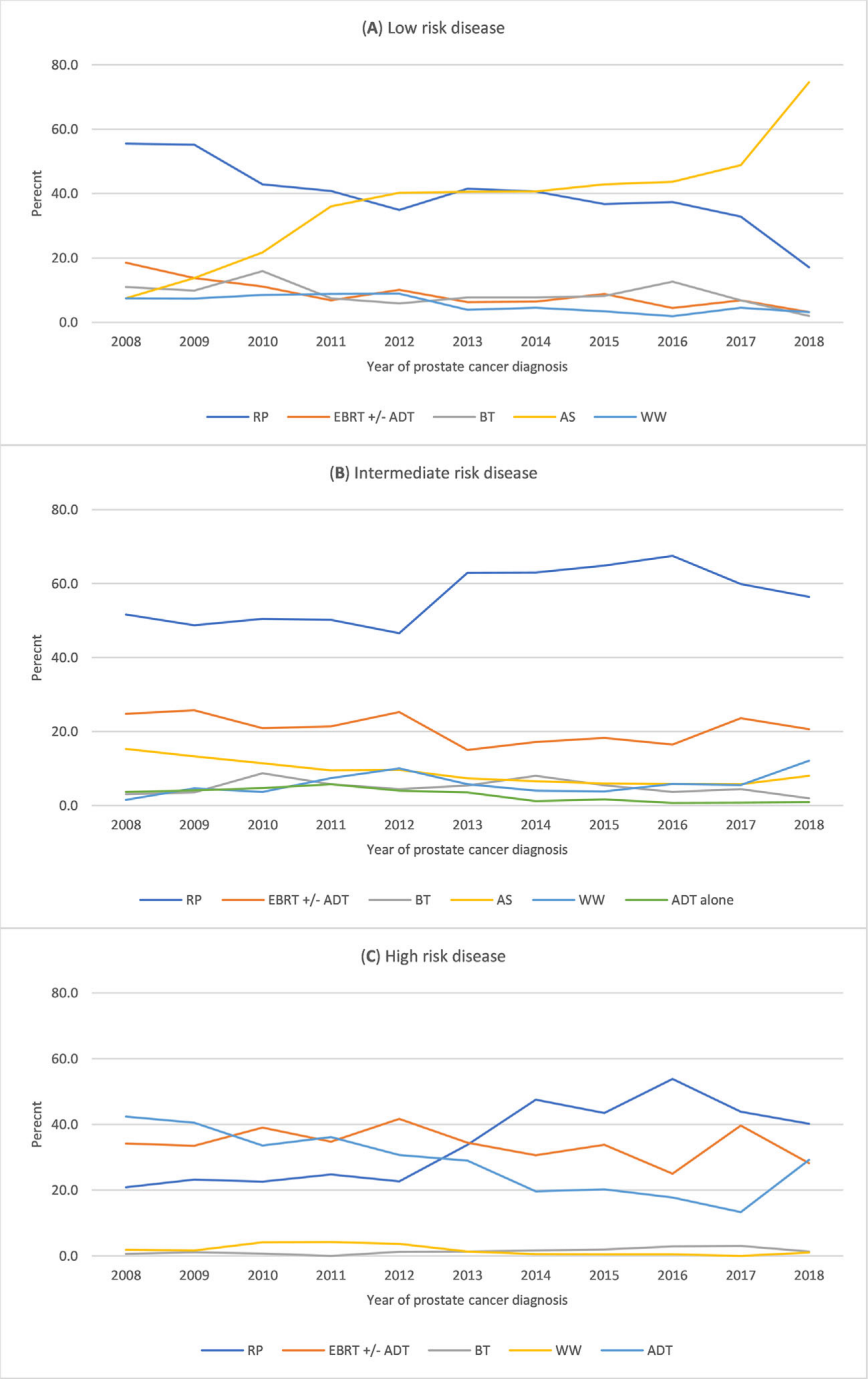
Treatment method stratified by risk group from 2008 to 2018, SA‐PCCOC registry.

In our study, only 38% of men with low‐risk disease were on active surveillance (Table S1). In the low‐risk group, proportion of men who underwent RP decreased overtime, while uptake of active surveillance increased from 7% in 2008 to 75% in 2018. Throughout the years, about half of men in the intermediate‐risk group underwent RP, peaking at 68% in 2016, whereas all other treatments remained relatively stable. Most men with high‐risk disease had either EBRT or ADT till 2013, but from 2013 onwards, RP became more popular. A decline in ADT was observed (from 42% in 2008 to 18% in 2017) among the high‐risk group (Figure 1).

### 3.1 Overall survival

Median follow‐up time was 7 years (interquartile range, 4–10), during which time 1298 patients had died including 430 from prostate cancer. Across all treatment options, the 5‐year overall survival probability for a 68‐year‐old man with low‐ or intermediate‐risk disease exceeded 91%. For high‐risk disease, the 5‐year survival probability ranged from 61% (95% CI: 46–78) among those on watchful waiting to 96% (95% CI: 95–98) following RP. Ten‐year overall survival probabilities were above 85% for low‐ and intermediate‐risk disease across all treatment options but varied more widely by treatment type for high‐risk disease (92%, 80%, 73% and 45% for RP, EBRT +/− ADT, ADT alone and watchful waiting, respectively) (Table 2).

**TABLE 2:** Probability of 5‐year and 10‐year median survival for the average 68‐year‐old man diagnosed with nonmetastatic prostate cancer, stratified by treatment type and risk category, SA‐PCCOC registry (men diagnosed 2008–2018).

Survival	Interval (years)^a^	Risk category^b^	RP			EBRT +/− ADT			BT^c^			AS^d^			WW			ADT		
			%	95% CI		%	95% CI		%	95% CI		%	95% CI		%	95% CI		%	95% CI	
Overall survival	5	Low	98.1	96.3	99.8	94.3	89.4	99.0	98.2	95.8	100	98.8	97.7	99.9	92.4	86.0	99.1	NA	—	—
		Intermediate	97.3	96.2	98.5	93.9	91.4	96.5	95.4	92.4	98.3	95.9	93.1	98.8	91.6	86.5	96.8	92.9	88.8	97.0
		High	96.4	94.8	98.1	85.9	81.9	89.9	—	—	—	NA	—	—	61.4	45.5	77.6	75.0	68.7	81.3
	10	Low	96.5	94.6	98.4	89.9	84.5	95.1	94.4	90.6	98.2	97.1	95.5	98.6	85.1	77.3	92.8	NA	—	—
		Intermediate	95.9	94.7	97.1	89.0	86.0	92.1	92.2	88.7	95.6	93.6	90.8	96.5	85.1	79.2	91.1	90.9	87.5	94.3
		High	91.8	89.2	94.3	80.0	75.5	84.3	—	—	—	NA	—	—	44.5	28.6	60.6	72.9	67.2	78.9
Prostate cancer‐specific survival	5	Low	99.8	99.6	100	99.1	98.2	100	99.6	99.1	100	100	—	—	98.7	97.3,	100	NA	—	—
		Intermediate	99.4	98.9	100	98.4	97.0	99.8	98.5	96.8	100	100	—	—	99.4	98.0	100	97.6	94.9	100
		High	98.8	97.8	99.7	92.3	89.4	95.6	—	—	—	NA	—	—	84.8	71.4	98.6	84.6	78.4	90.8
	10	Low	99.5	99.2	99.8	98.3	97.2	99.4	99.2	98.5	99.9	100	—	—	97.5	95.5	99.4	NA	—	—
		Intermediate	99.0	98.4	99.6	97.0	95.2	98.8	98.4	96.8	—	99.1	97.9	100	98.0	95.7	100	97.3	95.1	99.5
		High	96.7	95.0	98.3	88.8	85.1	92.6	—	—	—	NA	—	—	71.6	53.6	89.9	81.5	75.9	87.3

*Note*: Separate models (subgroup analyses) were executed for each risk category adjusted for age at diagnoses, year of diagnosis and socio‐economic advantage.

Abbreviations: ADT, androgen deprivation therapy alone; AS, active surveillance; BT, brachytherapy (low dose and high dose combined); CI, confidence interval; EBRT +/− ADT, external beam radiation therapy with or without ADT; NA, not applicable; RP, radical prostatectomy; WW, watchful waiting.

^a^ Time from primary treatment to either death or end of follow‐up.

^b^ Diagnostic PSA, Gleason score and clinical stage were imputed to generate risk category.

^c^ There were insufficient observation in the high‐risk group to estimate prostate cancer‐specific survival.

^d^ Men on active surveillance could have been switched to secondary treatments, and this has not been accounted in the analysis.

### 3.2 Prostate cancer‐specific survival

The 5‐year prostate cancer specific survival probability for a 68‐year‐old man commenced active surveillance was 100%. For high‐risk disease, 5‐year prostate cancer‐specific survival probabilities ranged from 85% (95% CI: 78–91) for ADT alone to 99% (95% CI: 98–100) for RP. Ten‐year prostate cancer‐specific survival probabilities for low‐ and intermediate‐risk disease were high (over 97%) across all treatment options. Ten‐year prostate cancer‐specific survival probabilities for high‐risk disease were 97%, 89%, 82% and 72% for RP, EBRT +/− ADT, ADT alone and watchful waiting, respectively (Table 2).

### 3.3 Biochemical recurrence

For a 68‐year‐old man with intermediate‐risk disease, the probability of developing a biochemical recurrence within 5 years was 17% (95% CI: 13–20) following RP, 13% (95% CI: 10–16) following EBRT +/− ADT and 10% (95% CI: 6–14) following brachytherapy. For high‐risk disease, the 5‐year probabilities of biochemical recurrence for a 68‐year‐old man were 37% (95% CI: 28–46) following RP and 17% (95% CI: 13–22) following EBRT +/− ADT. For high‐risk disease, biochemical recurrence only increased by 3% (from 37% at 5 years to 40% at 10 years) following RP, whereas increased by 10% (from 17% at 5 years to 27% at 10 years) following EBRT +/− ADT group (Table 3).

**TABLE 3:** Probability of developing biochemical recurrence for the average 68‐year‐old man diagnosed with nonmetastatic prostate cancer, stratified by treatment type and risk category, SA‐PCCOC registry (men diagnosed 2008–2018).

Interval (years)^a^	Risk category^b^	Radical prostatectomy			EBRT +/− ADT			BT		
		%	95% CI		%	95% CI		%	95% CI	
Two	Low	5.5	3.2	7.5	3.6	0.6,	6.4	2.4	0.3	4.5
	Intermediate	14.4	11.1	17.8	5.3	1.3	9.2	6.1	2.2	10.1
	High	31.0	24.6	37.5	8.8	3.6	12.8	NA	—	—
Five	Low	7.3	5.7	9.6	4.8	1.1	8.5	3.9	1.3	6.4
	Intermediate	16.8	13.4	20.2	12.9	9.5	16.4	9.9	5.6	13.8
	High	36.8	27.6	45.9	17.2	12.7	21.8	NA	—	—
Ten	Low	8.4	6.2	10.5	7.3	2.8	11.8	8.1	4.2	11.9
	Intermediate	18.4	14.8	22.1	17.6	7.6	27.7	12.9	6.5	19.3
	High	40.3	32.1	48.3	26.8	17.6	35.2	NA	—	—

*Note*: Prediction models were executed for each risk category adjusted for age at diagnoses, year of diagnosis and socio‐economic advantage.

Abbreviations: BT, brachytherapy; CI, confidence interval; EBRT+/‐ADT, external beam radiation therapy with or without ADT; NA, not applicable due to insufficient sample for meaningful analyses.

^a^ Time from primary treatment to either last follow‐up date or the date when the PSA test result defining the BCR was identified.

^b^ Diagnostic PSA, Gleason score and clinical stage were imputed to generate risk category.

### 3.4 Transition from active surveillance to active treatment

The probability of transitioning from active surveillance to active treatment within 2 years was 15% (95% CI: 10–20) with low‐risk prostate cancer and 20% (95% CI: 14–25) with intermediate‐risk prostate cancer. These increased to 27% (95% CI: 21–33) and 32% (95% CI: 26–38), respectively, by 5 years.

### 3.5 Patient‐reported outcome measures

Risk‐adjusted probabilities for having a clinically significant decline in physical functioning at 12 months after initial treatment, compared with baseline levels, are presented in Table 4 for each treatment option.

**TABLE 4:** Probability of having clinically significant decline in functional outcomes 1 year after treatment for the average 68‐years‐old man diagnosed with nonmetastatic prostate cancer, stratified by treatment type, SA‐PCCOC registry (men diagnosed 2008–2018).

Functional outcomes	RP			EBRT +/− ADT			BT			AS^a^			WW			ADT		
	%	95% CI		%	95% CI		%	95% CI		%	95% CI		%	95% CI		%	95% CI	
Sexual function	69.2	65.9	72.4	58.4	39.8	77.1	43.5	11.1	76.1	49.8	33.1	66.6	56.3	32.8	79.8	50.3	33.8	66.9
Urinary continence	57.1	53.2	60.9	30.9	20.3	40.5	28.1	13.0	43.2	35.5	26.9	44.1	33.8	9.4	58.1	25.4	8.6	42.2
Urinary irritation domain^b^	14.6	9.1	20.1	36.4	25.7	47.1	46.2	31.7	60.7	19.7	11.9	26.8	23.1	1.8	54.2	12.9	1.4	27.2
Bowel function	15.6	12.5	18.8	57.6	46.8	68.4	23.6	11.1	36.3	24.4	17.5	31.3	26.3	0.4	48.4	25.6	22.6	28.8
Hormonal function	31.7	28.3	35.1	48.6	31.0	66.4	26.5	12.7	40.5	12.1	5.3	18.9	17.1	3.6	30.6	56.8	45.3	68.4

*Note*: Decline in EPIC‐26 functional outcome scores at 12 months (from baseline score) were set at 10‐point score differences for sexual function, 6‐point for urinary continence, 5‐point for urinary irritation/obstruction domain and 4‐point for bowel and hormonal functions each. Prediction models were adjusted for inverse probability weighting, risk category, age at diagnoses, year of diagnosis and socio‐economic advantage. The analyses were not stratified by risk category due to inadequate observations for meaningful analyses.

Abbreviations: ADT, androgen deprivation therapy; AS, active surveillance; BT, brachytherapy; CI, confidence interval; EBRT +/− ADT, external beam radiation therapy with or without ADT; RP, radical prostatectomy; WW, watchful waiting.

^a^ Men on active surveillance could have been switched to secondary treatments, and this has not been accounted in the analysis.

^b^ Decline in urinary irritation domain score implies worse urinary irritative/obstructive problems.

For the average 68‐year‐old man, the probability of having clinically significant decline in sexual function following RP was 69% (95% CI: 66–72) with probabilities following other treatments ranging from 44% (95% CI: 11–76) for brachytherapy to 58% (95% CI: 40–77) for EBRT +/− ADT. The probability of experiencing a clinically significant decline in urinary continence was also highest following RP 57% (95% CI: 53–61) and was lowest following ADT 25% (95% CI: 9–42). Functional decline due to urinary obstruction/irritation was highest following brachytherapy, with probability of 46% (95% CI: 32–61), while declines in bowel function were greatest following EBRT +/− ADT, with probabilities of 57% (95% CI: 47–68). The probability of experiencing a clinically significant hormonal effect 12 months after starting ADT alone was 57% (95% CI: 45–68).

## 4 DISCUSSION

This study reports real‐world clinical and functional outcomes following each of the main primary treatment modalities for prostate cancer for a contemporary Australian cohort. Overwhelmingly, our study findings indicated favourable survival outcomes across most risk and treatment categories. Exceptions include men with high‐risk disease on either ADT or watchful waiting.

Given our study used real‐world data with inherent selection biases, we did not seek to directly compare outcomes between prostate cancer treatments. Rather, we have reported the probability of each outcome for the average man facing decisions about prostate cancer treatments. As such, our findings do not imply that men should choose one treatment over another, as each man's circumstances will differ.^3^ Clinician and patient considerations about treatment choices will depend on a range of factors including underlying medical conditions, cancer aggressiveness and extent of spread, personal circumstances and patient preferences. As reported previously,^20^ most men with localised prostate cancer die from causes other than their cancer. The presence of other comorbid conditions is likely to contribute to both poorer overall and poorer disease‐specific survival among men with high‐risk disease.^21^

Several findings from this real‐world data are consistent with results from the ProtecT trial including high prostate cancer‐specific survival rates,^22^ greatest decline of sexual function and urinary continence in prostatectomy group^2^ and worse bowel function in the radiotherapy group.^2^ Nevertheless, unlike our findings, the ProtecT trial showed less decline in functioning among the active monitoring group.

Favourable survival outcomes in a contemporary cohort are likely due to both earlier diagnosis and advances in treatment and management, including imaging technologies that enabled clinicians to more precisely stratify risk and recommend therapies based on cancer prognosis.^23^ The advancements in imaging and radical therapy techniques are likely to have led to improvements in functional and oncologic outcomes. For example, PSMA‐PET imaging improves the detection or staging of prostate cancer,^24^ whereas different nerve‐sparing surgical techniques^25^, ^26^, ^27^ and more accurate delivery of radiotherapies such as intensity‐modulated radiation therapy (IMRT) and image‐guided radiation therapy (IGRT) are likely to have led to an improvement in functioning after treatment.^28^, ^29^ Advancements in multimodal therapies are expected to further improve survival, particularly for men with high‐risk disease.

Our results relating to biochemical recurrence are in line with the findings of the systematic review by Fakhrejahani et al^30^ report that about one‐third of men on RP and 25%–33% of men on radiotherapy develop biochemical recurrence within 5 years. Conversely, a more recent report indicates a significantly higher risk of biochemical recurrence for patients receiving EBRT compared to RP 10 years after treatment, and the cumulative incidence of biochemical recurrence was 23.8% (95% CI: 17.9–30.2) in the RP and 43.0% (95% CI: 34.9–50.8) in the EBRT groups.^31^ Our findings regarding biochemical recurrence should be interpreted with caution. Differences between the RP and the EBRT +/− ADT groups are likely the higher proportion who remain on ADT after radiotherapy, which lowers PSA levels,^32^ leading to an underestimate of biochemical recurrence in the EBRT +/− ADT group. In addition, it is difficult to directly compare treatment modalities due to different (standard) definitions of biochemical recurrence for RP and radiotherapy and potentially differences in the frequency of post‐treatment PSA testing. Furthermore, our findings in the EBRT +/− ADT group may reflect the lower risk of biochemical recurrence associated with the current trend towards the use of dose‐escalated radiotherapy, as reported by Tanaka et al.^33^ While the relationship between biochemical recurrence and prostate cancer mortality remains unclear, biochemical recurrence is an important trigger for secondary treatment (e.g. salvage radiotherapy/RP or ADT) with associated morbidity.

Our results relating to patient reported outcomes clearly show that all treatment modalities negatively impacted physical functioning, although the domain‐specific effects were varied between the treatments. Compared to other studies,^34^, ^35^ we reported quite high levels of impact for most functional outcomes. This is, in part, due to choosing to report the likelihood of any ‘clinically significant decline’, defined as the smallest differences that patients can notice.^17^ Hence, our results are indicative of the impact on functional outcomes that could be problematic from a patient's perspective.

In this study, RP remained most commonly used treatment for men with intermediate‐ and high‐risk groups (2013 onwards), while the use of radiotherapies was relatively steady across the years. Recent evidence from analyses of the Australian Medicare Benefits Schedule data has shown that the use of RP increased between 2002 and 2009, but from 2012 to 2016, the rates of RP (15% drop) subsequently decreased, while the use of EBRT remained steady.^36^ We have also observed an increased trend of active surveillance uptake by men with low‐risk prostate cancer. This is in line with reports from the binational registry (PCOR‐ANZ) where the proportion of men on active surveillance has increased over time (54% in 2015 to 71% in 2018), while radical treatment rates have decreased.^37^ Active surveillance is recommended for men diagnosed with low risk and some favourable intermediate‐risk localised prostate cancer, with an aim to reduce overtreatment and minimise the negative side effects of radical therapies while preserving quality of life, ensuring the option for curative treatment, without losing the window of curability.^38^, ^39^

The strength of this study is its use of ‘real‐world data’ that reflects the actual experience of men diagnosed with prostate cancer. Our use of a decade worth of data that collects baseline PROMs is a contemporary addition to existing evidence. PROMs were reported based on ‘any’ clinically significant decline, which allows interpretation of the data from patient perspectives. We have used advanced statistical models standardised for risk classification and age, have accounted for missingness (imputed variables defining risk) and have applied propensity weighting to adjust for missing EPIC items. SA‐PCCOC is a community‐wide registry incorporating a heterogeneous patient population including those managed in large tertiary centres as well as smaller private practices and hence can provided a population‐wide picture of outcomes for prostate cancer. Population‐based prospective disease‐specific registries have value in influencing clinical practice and improving outcomes overtime. They provide important information on mortality, disease characteristics at presentation, long‐term oncologic and quality of life outcomes as well as quality of care.^40^ Two recent studies using the binational prostate cancer registry (PCOR‐ANZ) showed population‐level data demonstrated clinically equivalent PROMs from randomised controlled trials.^41^, ^42^ Another study using PCOR‐Vic revealed that positive margin rate has significantly reduced since the inception of the registry, where the authors commented that benchmarking against established aspirational real‐world post‐operative outcomes facilitates the detection of areas of improvement.^43^ Further improvements in data quality will increase the relevance of studies derived from such registries. SA‐PCCOC as a main contributor to the PCOR‐ANZ, evidence on risk‐adjusted posttreatment outcomes would leverage other registries and provide benchmarks to encourage quality improvement efforts to produce improved outcomes.

Limitations include that we did not account for secondary treatments in our assessment of outcomes, due to incomplete data on additional treatments. Adjuvant and salvage therapies were not accounted for due to practices vary widely among treating clinicians regarding salvage and adjuvant therapies, and data were not consistently collected. The receipt of adjuvant or salvage therapy with radical therapies could affect outcomes; for example, the decline in PROMs among men who had EBRT +/− ADT is likely to be worse than among men who had EBRT alone. In this study, we reported the outcomes for EBRT as the primary treatment group including ADT with EBRT, so reflects the real‐world experiences among men receiving EBRT according to current best practice. Another limitation is that the type of radical treatment technique was not separately compared, and the analyses did not account surgical margins status, which may influence the outcomes. Furthermore, there may be misclassification regarding observant management, given some inconsistency in recording active surveillance and watchful waiting as distinct approaches during the earlier period of this study. Men were categorised as having active surveillance if this was recorded as their initial treatment if stated in their medical record or if not specifically stated through application of an algorithm based on risk level and clinical characteristics. Also, we only present functional changes at 12 months and not at longer intervals since PROMs are routinely captured at 12 months and 24‐month measures are no longer collected by SA‐PCCOC. Previous (unpublished) analysis of SA data shows little improvement in functional scores between 12 and 24 months. Hence, we believe that our data are indicative of the likely impact on men's physical function.

## 5 CONCLUSION

Both clinical and functional outcomes need to be considered when choosing between treatment options to manage prostate cancer. This study reports on multiple clinical and functional outcomes following different treatments for an Australian cohort, based on real‐world experience. Findings indicate that survival was generally high across all treatments. However, the impact of treatments on physical functioning varied considerably. Notably, declines in sexual function occur across all treatment approaches, but most frequently following RP. Discussions with patients and their families about post‐treatment functional declines could minimise/avoid regret due to unrealistic expectations.

## AUTHOR CONTRIBUTIONS

Kerri Beckmann, Tenaw Tiruye, Michael O'Callaghan, Kerry Ettridge, Kim Moretti and Kerry Santoro conceived and designed the research; Tenaw Tiruye, Kerri Beckmann and Michael O'Callaghan analysed the data; Tenaw Tiruye and Kerri Beckmann wrote the original draft; Michael O'Callaghan, Kerry Ettridge, Kim Moretti, Alex Jay, Braden Higgs, Ganessan Kichenadasse and Kerry Santoro reviewed and edited the manuscript. All authors have read and approved the submitted version of the manuscript.

## CONFLICT OF INTEREST STATEMENT

No conflicts of interest.

## Supporting information


**Table S1:** Proportion of men with low‐risk diseases who went onto active surveillance.

## References

[1] Australian Institute of Health and Welfare . Australian cancer incidence and mortality (ACIM) book: prostate cancer. Canbera; 2021. Available from: https://www.aihw.gov.au/reports/cancer/cancer-data-in-australia/data

[2] Donovan JL , Hamdy FC , Lane J , Mason M , Metcalfe C , Walsh E , et al. Patient‐reported outcomes after monitoring, surgery, or radiotherapy for prostate cancer. N Engl J Med. 2016;375(15):1425–1437. 10.1056/NEJMoa1606221 27626365 PMC5134995

[3] Cancer Council Australia . Understanding prostate cancer: a guide for people with cancer, their families and friends. Sydney, NSW March 2022. 76 p.

[4] The National Institute for Health and Care Excellence (NICE) . NICE guidance – prostate cancer: diagnosis and management. BJU Int. 2019;124(1):9–26. 10.1111/bju.14809 31206997

[5] Kinsella N , Helleman J , Bruinsma S , Carlsson S , Cahill D , Brown C , et al. Active surveillance for prostate cancer: a systematic review of contemporary worldwide practices. Transl Androl Urol. 2018;7(1):83–97. 10.21037/tau.2017.12.24 29594023 PMC5861285

[6] Ziehr DR , Chen MH , Zhang D , Braccioforte MH , Moran BJ , Mahal BA , et al. Association of androgen‐deprivation therapy with excess cardiac‐specific mortality in men with prostate cancer. BJU Int. 2015;116(3):358–365. 10.1111/bju.12905 25124891

[7] Zelic R , Garmo H , Zugna D , Stattin P , Richiardi L , Akre O , et al. Predicting prostate cancer death with different pretreatment risk stratification tools: a head‐to‐head comparison in a nationwide cohort study. Eur Urol. 2020;77(2):180–188. 10.1016/j.eururo.2019.09.027 31606332

[8] Smith DP , King MT , Egger S , Berry MP , Stricker PD , Cozzi P , et al. Quality of life three years after diagnosis of localised prostate cancer: population based cohort study. BMJ (Clinical Research Ed). 2009;339(nov27 2):b4817. 10.1136/bmj.b4817 PMC2784818 19945997

[9] Di Maio M , Perrone F , Conte P . Real‐world evidence in oncology: opportunities and limitations. Oncologist. 2020;25(5):e746–e752. 10.1634/theoncologist.2019-0647 31872939 PMC7216461

[10] Royston P , White IR . Multiple imputation by chained equations (MICE): implementation in Stata. J Stat Softw. 2011;45(4):1–20. 10.18637/jss.v045.i04

[11] Mohler JL , Antonarakis ES , Armstrong AJ , D'Amico AV , Davis BJ , Dorff T , et al. Prostate cancer, version 2.2019, NCCN clinical practice guidelines in oncology. J Natl Compr Canc Netw. 2019;17(5):479–505. 10.6004/jnccn.2019.0023 31085757

[12] Australian Bureau of Statistics . Census of population and housing: socio‐economic indexes for areas (SEIFA), Australia, 2016 Australia. ABS Website; 2018. Available from: https://www.abs.gov.au/ausstats/abs@.nsf/mf/2033.0.55.001

[13] International Consortium for Health Outcomes Measurement . Patient‐centred outcome measures for localized prostate cancer 2017. Available from: https://connect.ichom.org/patient-centered-outcome-measures/localized-prostate-cancer/

[14] Stephenson AJ , Kattan MW , Eastham JA , Dotan ZA , Bianco FJ Jr , Lilja H , et al. Defining biochemical recurrence of prostate cancer after radical prostatectomy: a proposal for a standardized definition. J Clin Oncol. 2006;24(24):3973–3978. 10.1200/JCO.2005.04.0756 16921049

[15] Roach M 3rd , Hanks G , Thames H Jr , Schellhammer P , Shipley WU , Sokol GH , et al. Defining biochemical failure following radiotherapy with or without hormonal therapy in men with clinically localized prostate cancer: recommendations of the RTOG‐ASTRO Phoenix consensus conference. Int J Radiat Oncol Biol Phys. 2006;65(4):965–974. 10.1016/j.ijrobp.2006.04.029 16798415

[16] Wei JT , Dunn RL , Litwin MS , Sandler HM , Sanda MG . Development and validation of the expanded prostate cancer index composite (EPIC) for comprehensive assessment of health‐related quality of life in men with prostate cancer. Urology. 2000;56(6):899–905. 10.1016/S0090-4295(00)00858-X 11113727

[17] Skolarus TA , Dunn RL , Sanda MG , Chang P , Greenfield TK , Litwin MS , et al. Minimally important difference for the expanded prostate cancer index composite short form. Urology. 2015;85(1):101–105. 10.1016/j.urology.2014.08.044 25530370 PMC4274392

[18] Lambert PC , Andersson TML , Rutherford MJ , Myklebust TÅ , Møller B . Reference‐adjusted and standardized all‐cause and crude probabilities as an alternative to net survival in population‐based cancer studies. Int J Epidemiol. 2020;49(5):1614–1623. 10.1093/ije/dyaa112 32829393

[19] Seaman SR , White IR . Review of inverse probability weighting for dealing with missing data. Stat Methods Med Res. 2013;22(3):278–295. 10.1177/0962280210395740 21220355

[20] Riihimäki M , Thomsen H , Brandt A , Sundquist J , Hemminki K . What do prostate cancer patients die of? Oncologist. 2011;16(2):175–181. 10.1634/theoncologist.2010-0338 21257717 PMC3228081

[21] Hall WH , Jani AB , Ryu JK , Narayan S , Vijayakumar S . The impact of age and comorbidity on survival outcomes and treatment patterns in prostate cancer. Prostate Cancer Prostatic Dis. 2005;8(1):22–30. 10.1038/sj.pcan.4500772 15700051

[22] Hamdy FC , Donovan JL , Lane JA , Mason M , Metcalfe C , Holding P , et al. 10‐year outcomes after monitoring, surgery, or radiotherapy for localized prostate cancer. N Engl J Med. 2016;375(15):1415–1424. 10.1056/NEJMoa1606220 27626136

[23] Litwin MS , Tan H‐J . The diagnosis and treatment of prostate cancer: a review. Jama. 2017;317(24):2532–2542. 10.1001/jama.2017.7248 28655021

[24] Maurer T , Eiber M , Schwaiger M , Gschwend JE . Current use of PSMA–PET in prostate cancer management. Nat Rev Urol. 2016;13(4):226–235. 10.1038/nrurol.2016.26 26902337

[25] Cochetti G , Del Zingaro M , Ciarletti S , Paladini A , Felici G , Stivalini D , et al. New evolution of robotic radical prostatectomy: a single center experience with PERUSIA technique. Appl Sci. 2021;11(4):1513. 10.3390/app11041513

[26] Lee J , Kim HY , Goh HJ , Heo JE , Almujalhem A , Alqahtani AA , et al. Retzius sparing robot‐assisted radical prostatectomy conveys early regain of continence over conventional robot‐assisted radical prostatectomy: a propensity score matched analysis of 1,863 patients. J Urol. 2020;203(1):137–144. 10.1097/JU.0000000000000461 31347951

[27] Haga N , Miyazaki T , Tsubouchi K , Okabe Y , Shibayama K , Emoto D , et al. Comprehensive approach for preserving cavernous nerves and erectile function after radical prostatectomy in the era of robotic surgery. Int J Urol. 2021;28(4):360–368. 10.1111/iju.14491 33508871

[28] Yu T , Zhang Q , Zheng T , Shi H , Liu Y , Feng S , et al. The effectiveness of intensity modulated radiation therapy versus three‐dimensional radiation therapy in prostate cancer: a meta‐analysis of the literatures. PLoS ONE. 2016;11(5):e0154499. 10.1371/journal.pone.0154499 27171271 PMC4865138

[29] Zapatero A , Roch M , Büchser D , Castro P , Fernández‐Banda L , Pozo G , et al. Reduced late urinary toxicity with high‐dose intensity‐modulated radiotherapy using intra‐prostate fiducial markers for localized prostate cancer. Clin Transl Oncol. 2017;19(9):1161–1167. 10.1007/s12094-017-1655-9 28374321

[30] Fakhrejahani F , Madan RA , Dahut WL . Management options for biochemically recurrent prostate cancer. Curr Treat Options Oncol. 2017;18(5):26. 10.1007/s11864-017-0462-4 28434181

[31] Suárez JF , Zamora V , Garin O , Gutiérrez C , Pont À , Pardo Y , et al. Mortality and biochemical recurrence after surgery, brachytherapy, or external radiotherapy for localized prostate cancer: a 10‐year follow‐up cohort study. Sci Rep. 2022;12(1):12589. 10.1038/s41598-022-16395-w 35869124 PMC9307750

[32] Sasaki T , Sugimura Y . The importance of time to prostate‐specific antigen (PSA) nadir after primary androgen deprivation therapy in hormone‐Naïve prostate cancer patients. J Clin Med. 2018;7(12):565. 10.3390/jcm7120565 30567361 PMC6306761

[33] Tanaka N , Asakawa I , Katayama E , Hirayama A , Hasegawa M , Konishi N , et al. The biochemical recurrence‐free rate in patients who underwent prostate low‐dose‐rate brachytherapy, using two different definitions. Radiat Oncol. 2014;9(1):107. 10.1186/1748-717X-9-107 24885896 PMC4029825

[34] Chen RC , Basak R , Meyer A‐M , Kuo T‐M , Carpenter WR , Agans RP , et al. Association between choice of radical prostatectomy, external beam radiotherapy, brachytherapy, or active surveillance and patient‐reported quality of life among men with localized prostate cancer. Jama. 2017;317(11):1141–1150. 10.1001/jama.2017.1652 28324092 PMC6284802

[35] Nguyen‐Nielsen M , Moller H , Tjonneland A , Borre M . Patient‐reported outcome measures after treatment for prostate cancer: results from the Danish prostate cancer registry (DAPROCAdata). Cancer Epidemiol. 2020;64:101623. 10.1016/j.canep.2019.101623 31760356

[36] Roberts MJ , Papa N , Perera M , Scott S , Teloken PE , Joshi A , et al. A contemporary, nationwide analysis of surgery and radiotherapy treatment for prostate cancer. BJU Int. 2019;124(S1):31–36. 10.1111/bju.14773 31486575

[37] Papa N , O'Callaghan M , James E , Millar J . Prostate cancer in Australian and New Zealand men: patterns of care within PCOR‐ANZ 2015–2018. Melbourne, VIC: Monash University & Movember; 2021.

[38] Briganti A , Fossati N , Catto JWF , Cornford P , Montorsi F , Mottet N , et al. Active surveillance for low‐risk prostate cancer: the European Association of Urology position in 2018. Eur Urol. 2018;74(3):357–368. 10.1016/j.eururo.2018.06.008 29937198

[39] Salari K , Kuppermann D , Preston MA , Dahl DM , Barrisford GW , Efstathiou JA , et al. Active surveillance of prostate cancer is a viable option for men younger than 60 years. J Urol. 2019;201(4):721–727. 10.1097/JU.0000000000000031 30664083

[40] Gandaglia G , Bray F , Cooperberg MR , Karnes RJ , Leveridge MJ , Moretti K , et al. Prostate cancer registries: current status and future directions. Eur Urol. 2016;69(6):998–1012. 10.1016/j.eururo.2015.05.046 26056070

[41] O'Callaghan ME , Roberts MJ , Moretti KL , Frydenberg M , Gilbourd D , Mark S , et al. Variation in patient reported outcomes following radical prostatectomy: a bi‐national registry‐based study. Urol Oncol: Sem Orig Invest. 2023;41(2):105.e9–105.e18. 10.1016/j.urolonc.2022.10.020 36437157

[42] Pryor DI , Martin JM , Millar JL , Day H , Ong WL , Skala M , et al. Evaluation of hypofractionated radiation therapy use and patient‐reported outcomes in men with nonmetastatic prostate cancer in Australia and New Zealand. JAMA Netw Open. 2021;4(11):e2129647. 10.1001/jamanetworkopen.2021.29647 34724555 PMC8561328

[43] Papa N , Perera M , Bensley JG , Evans M , Millar J , Frydenberg M , et al. A decade of declining prostatectomy margin positivity within a prostate cancer clinical quality registry. Urol Oncol: Sem Orig Invest. 2022;40(12):537.e19–537.e24. 10.1016/j.urolonc.2022.08.012 36167774

