# Supplementary material for: Clinical and functional outcomes for risk‐appropriate treatments for prostate cancer
Source: BJUI Compass. 2023 Sep 12;5(1):109–20. doi: 10.1002/bco2.288 (PMC10764171; doi:10.1002/bco2.288)
Supplement: Supplementary file 1 — Table S1: Proportion of men with low‐risk diseases who went onto active surveillance. [file BCO2-5-109-s001.docx]

Supplementary Table 1: Proportion of men with low-risk diseases who went onto active surveillance

| Year of diagnosis | Men on AS | Men with low-risk disease | Percent |
| --- | --- | --- | --- |
| 2008 | 15 | 206 | 7.3 |
| 2009 | 28 | 204 | 13.7 |
| 2010 | 41 | 190 | 21.6 |
| 2011 | 53 | 147 | 36.1 |
| 2012 | 68 | 169 | 40.2 |
| 2013 | 84 | 208 | 40.4 |
| 2014 | 63 | 155 | 40.6 |
| 2015 | 63 | 147 | 42.9 |
| 2016 | 69 | 160 | 43.1 |
| 2017 | 107 | 219 | 48.9 |
| 2018 | 188 | 252 | 74.6 |
| Total | 779 | 2,057 | 37.9 |
